# Host-response testing with MeMed BV in community-acquired pneumonia: an economic evaluation from the UK NHS perspective

**DOI:** 10.1093/jacamr/dlaf016

**Published:** 2025-02-19

**Authors:** Emily Gregg, Sara Graziadio, William Green, Daniela Afonso, Monica Garrett, Karina Watts, Deborah Watkins, Enitan D Carrol, Jonathan Cooke, Tim Felton

**Affiliations:** York Health Economics Consortium (YHEC), University of York, York, UK; York Health Economics Consortium (YHEC), University of York, York, UK; York Health Economics Consortium (YHEC), University of York, York, UK; York Health Economics Consortium (YHEC), University of York, York, UK; York Health Economics Consortium (YHEC), University of York, York, UK; York Health Economics Consortium (YHEC), University of York, York, UK; York Health Economics Consortium (YHEC), University of York, York, UK; Department of Clinical Infection, Microbiology and Immunology, University of Liverpool Institute of Infection, Veterinary and Ecological Sciences, Liverpool, UK; Department of Paediatric Infectious Diseases and Immunology, Alder Hey Children’s NHS Foundation Trust, Liverpool, UK; NIHR London In Vitro Diagnostics Co-operative, Division of Surgery, Department of Surgery and Cancer, Faculty of Medicine, Imperial College, London, UK; Manchester Pharmacy School, Faculty of Biology, Medicine and Health, University of Manchester, Manchester, UK; Division of Immunology, Immunity to Infection and Respiratory Medicine, Faculty of Biology, Medicine and Health, University of Manchester, Manchester, UK; Wythenshawe Hospital, Manchester University NHS Foundation Trust, Manchester, UK

## Abstract

**Background:**

Community-acquired pneumonia (CAP) remains a leading cause of hospital admissions and mortality. A novel host-response test, MeMed BV (MMBV), has been developed for discriminating between bacterial and viral infection that could improve the clinical management of CAP.

**Objectives:**

To evaluate the cost-effectiveness of using MMBV to guide antibiotic decisions in the clinical management of CAP in the UK.

**Methods:**

An economic model was developed to understand the incremental cost per person associated with the implementation of MMBV from the UK NHS perspective. A qualitative care pathway analysis was performed to inform the standard of care (SOC) and SOC plus MMBV (SOC + MMBV) clinical pathways captured in the model.

**Results:**

In the base case analysis, the SOC + MMBV strategy for a hypothetical cohort of 1000 patients (adults and children modelled independently) presenting to the emergency department with suspected CAP was estimated to provide total cost savings of £134 018 and £105 750 for adults and children, respectively. Cost savings were associated with reductions in total antibiotic treatment, the number of patients receiving additional diagnostic tests, and hospital admissions. Deterministic sensitivity analysis revealed that the specificity of SOC + MMBV and sensitivity of the SOC were primary drivers of the cost model for adults, whereas the specificity of SOC and SOC + MMBV were primary drivers for paediatrics.

**Conclusions:**

Overall, the model predicts that the introduction of SOC + MMBV has the potential to be cost-saving and promote antimicrobial stewardship for both adult and paediatric CAP patients.

## Introduction

Community-acquired pneumonia (CAP) remains a tremendous clinical challenge and is associated with a high healthcare burden and excess mortality.^1,2^ Overestimating the probability of bacterial CAP leads to inappropriate antibiotic use,^3^ which exacerbates the problem of antimicrobial resistance. Recent epidemiological data, however, indicate an equal or even higher contribution of viral aetiology in CAP.^4–6^ Despite the available evidence, many clinical guidelines still recommend prompt antibiotic use irrespective of infectious aetiology.^7,8^ Therefore, improving the diagnosis of bacterial- and viral-associated CAP is needed.

In the last decade, a plethora of novel technologies have become available to aid with the aetiological diagnosis of CAP. Pathogen-based testing strategies have been implemented in the emergency department (ED) to improve antimicrobial use. Yet, recent systematic reviews and meta-analyses revealed that routine rapid viral testing was primarily associated with higher influenza antiviral use.^9,10^ One possible explanation for these findings is clinician hesitation to avoid prescribing antibiotics for viral infections, given that up to 40% of such cases may involve bacterial coinfections.^11^ Beside pathogen-based testing strategies, inflammatory biomarkers, such as C-reactive protein (CRP) and procalcitonin (PCT), are widely incorporated into routine clinical practice. However, conflicting data on their added benefit in managing respiratory infections limit their applicability to inform antimicrobial prescribing.^12,13^ Accordingly, the two recent clinical guidelines prompted only a weak recommendation for CRP and PCT to inform antibiotic use in patients with respiratory tract infections. One promising novel tool that has emerged combines the measurement of three host-protein biomarkers—TNF-related apoptosis-inducing ligand (TRAIL), interferon-γ-induced protein-10 (IP-10) and CRP—into a bacterial versus viral likelihood score to improve the judicious use of antibiotics (antimicrobial stewardship).^14^ This test, called MeMed BV (MMBV), has demonstrated good diagnostic accuracy for differentiating bacterial from viral infections in multiple studies.^15–17^ In the multicentre MMBV FDA-clearance trial conducted in the USA and Israel, MMBV had a sensitivity (i.e. the number of bacterial infections that were correctly diagnosed) of 90% (95% CI: 80.3%–99.7%) and specificity (i.e. the number of viral infections that were correctly diagnosed) of 92.8% (95% CI: 90.0%–95.5%).^17^ The diagnostic accuracy of MMBV was evaluated using a reference standard based on clinical adjudication of comprehensive case information, incorporating all available clinical or diagnostic evidence available.^18^

Successful implementation of novel testing strategies for the management of CAP requires robust evidence related to the efficacy and cost-effectiveness of the diagnostic to inform wider applicability in the healthcare system. Therefore, an early-stage economic evaluation of the MMBV test integrated into the CAP clinical pathway in the UK was performed. Additionally, integration of a qualitative care pathway analysis (CPA) with existing diagnostic accuracy data was recently proposed as the optimal strategy to inform diagnostic cost-effectiveness models.^19^

For this reason, a qualitative CPA was undertaken to map the standard of care (SOC) and SOC plus MMBV (SOC +MMBV) clinical pathways that were incorporated into the economic model.

## Materials and methods

### Ethics

Ethical approval was obtained from the Health Sciences Research Governance committee at the University of York (reference: HSRGC/2023/576/A). Written informed consent was obtained from the interviewees in the CPA. Confidentiality and anonymity were maintained throughout this research.

### Care pathway analysis

A qualitative evaluation, using semi-structured interviews with relevant stakeholders (i.e. clinicians and payers) and thematic analysis, was conducted before developing the economic model. The CPA research questions and methods were predefined in a protocol. The detailed methodological approach for conducting CPA has been described previously.^20,21^ Briefly, 60 min semi-structured interviews were conducted with 14 experts in: (i) management and diagnosis of lower respiratory tract infection (LRTI)/CAP, (ii) diagnostic test adoption, and (iii) procurement in the NHS. All interviewees were based in the UK. A description of the interviewees’ characteristics is reported in Table S1 (available as Supplementary data at *JAC-AMR* Online).

A topic guide was created, including questions to identify the SOC and SOC + MMBV pathways and a draft care pathway flow diagram. The draft care pathway was developed based on published guidance.^22,23^ The topic guide was tailored to account for the two subgroups of interviewees: ‘clinicians’ and ‘budget stakeholders’. The ‘clinician’ topic guide is provided in Appendix S1. Interviews were conducted between July and August 2023. Two researchers (S.G. and E.G.) trained in qualitative research conducted the interviews. Interviews were analysed thematically by one researcher (E.G./D.W./K.W.) and validated by the second researcher (S.G.).

### Model overview

The economic evaluation is reported using the CHEERS criteria and was aligned with the National Institute for Health and Care Excellence methods manual.^24^ A completed CHEERS criteria checklist can be found in Table S2. Costs were applied from the perspective of the English NHS and Personal Social Services. Outcomes were quantified in terms of number of correct/incorrect viral and bacterial infections. The economic model was built in Microsoft Excel and focused on generating incremental cost estimates of SOC + MMBV over SOC alone. The time horizon was less than 1 year. A hypothetical cohort of 1000 patients with suspected CAP presenting to the ED was modelled. Adults and children were modelled independently. Primary cost drivers were based on the themes identified in the CPA and included: antibiotic stewardship, diagnostic stewardship, admission avoidance and readmission elements. A separate scenario for adult and paediatric CAP was created.

### Model structure

**Figure 1.**
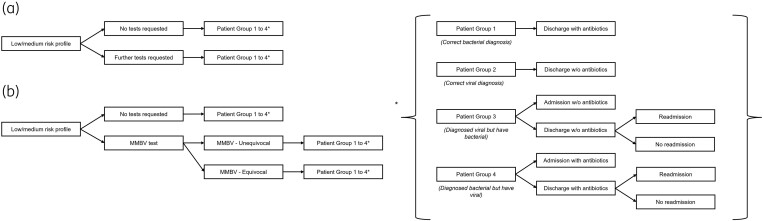
Economic decision trees for SOC (a) and SOC + MMBV (b). All patients entered the model with suspected CAP and presenting to the ED. For both SOC and SOC + MMBV, the model cohorts were split by a clinical decision of whether tests were required and, for the SOC + MMBV cohort, whether an MMBV test was required. Clinical outcome parameters investigated are demonstrated in braces. w/o, without.

A decision-tree structure (Figure 1) was used to capture the total costs of diagnosing and treating the hypothetical cohort of 1000 patients with suspected CAP presenting to the ED of a typical NHS hospital. Patients entered the model with a low/medium risk profile as per the National Early Warning Score 2/Paediatric Early Warning System (adults/children) assessment defined in the CPA. These patients were identified as more likely candidates for withholding antibiotics or benefiting from diagnostic tools to guide antibiotic stewardship, whereas high-risk patients typically require immediate treatment decisions, including hospitalization. This assumption was based on the output of the CPA. In the decision tree, patients were split into two groups based on whether any test would be used to inform the initial treatment decision. This decision was guided by clinician interviews and supporting literature, ensuring the model reflected real-world clinical practice.^15,16,25^ Subsequently, patients were classified into four categories, namely: correctly diagnosed viral illness, correctly diagnosed bacterial illness, misclassified viral illness, and misclassified bacterial illness. Correct viral diagnosis implied no antibiotics and patients could be discharged with no further treatment. Correct bacterial diagnosis implied appropriate antibiotics were administered and patients could be discharged with no long-term care required. Misclassified bacterial infections included patients that were not administered an antibiotic when they should have been. Subsequently, their condition deteriorated before antibiotics were correctly given. A proportion of these patients required hospital admission due to the deterioration. Misclassified viral infections included patients incorrectly administered antibiotics and, due to misclassification, it was assumed that a proportion of these patients were admitted to an observation ward.

The probability of each classification category was based on the diagnostic accuracy of SOC or SOC + MMBV. Further details on the reference standard and methodology underlying the diagnostic process can be found in a recent publication.^18^ For SOC + MMBV, equivocal MMBV score results (from 35 to 65) would be classified into the four categories based on the diagnostic accuracy of SOC alone. Modelling assumptions were introduced to ensure the model design was not overly complex. These included: (i) all correctly diagnosed viral patients could be discharged with no further treatment, (ii) all correctly diagnosed bacterial patients could be discharged following administration of antibiotics only, and (iii) neither of these groups would require long-term care. In reality, a proportion of these patients may deteriorate and require hospital admission. However, the risk was expected to be sufficiently small that such events could be excluded from the model.

### Scenarios

The model’s functionality was calibrated to explore the scenario of costs associated with bacterial-viral coinfections. Bacterial-viral coinfections were excluded from the base case analysis because accurately defining such coinfections in current clinical practice remains challenging, as pathogen detection does not necessarily imply causation. Additionally, the introduction of host-response–based testing is likely to advance our understanding of coinfections. Therefore, coinfections were analysed as a scenario to avoid introducing additional assumptions and reduce the generalizability of the base model output. In the bacterial-viral coinfection scenario, patients in the SOC + MMBV arm entered the model with a coinfection and subsequently received the MMBV test. Patients could either have no coinfection or a coinfection. The latter group was split depending on whether the coinfection was detected or not. Those patients in the SOC arm also entered the model with a coinfection and received further diagnostic testing. Patients were then split depending on whether a coinfection was detected or not.

### Model inputs

#### Clinical decision making

The baseline inputs on clinical decision making are summarized in Table 1. Estimates of infectious aetiology in CAP, the proportion of patients receiving diagnostic tests, MMBV and SOC + MMBV diagnostic accuracy, and MMBV score distribution were available from published literature.^4,5,15,16,26^ The proportion of patients receiving a diagnostic work-up was based on a prospective observational study that analysed clinician decision making in patients with suspected CAP using vignettes.^25^

**Table 1.** Base case clinical decision inputs

		SOC	SOC + MMBV	Reference
**I. Infectious aetiology in CAP**				
Adults	% Bacterial	31.79		^5^
	% Viral	68.21		
Children	% Bacterial	10.63		^4^
	% Viral	89.37		
**II. Proportion of patients with diagnostic workup**				
% Test ordered		66.76		^25^
% No tests ordered		33.24		
**III. MMBV score distribution**				
Adults	% Bacterial	—	45.78	^16^
	% Viral	—	44.10	
	% Equivocal	—	10.12	
Children	% Bacterial	—	16.67	^15^
	% Viral	—	73.50	
	% Equivocal	—	9.84	
**IV. Diagnostic accuracy estimates**				
Adults	Sensitivity	98.08%	98.98%	^16^
	Specificity	44.29%	81.43%	
Children	Sensitivity	91.35%	93.27%	^15^
	Specificity	70.38%	90.92%	
**V. Detection rates**				
Adults	True positives	31.20%	31.20%	
	False negatives	0.60%	0.60%	
	False positives	38.00%	12.7%	
	True negatives	30.20%	55.50%	
Children	True positives	9.70%	9.90%	
	False negatives	0.90%	0.70%	
	False positives	26.5%	8.10%	
	True negatives	62.90%	81.30%	
**VI. Coinfection scenario**				
Adults	Viral PCR positive, bacterial outcome	11.06%		^15,16^
	Bacterial-viral coinfection detection rate	96.00%		
Children	Viral PCR positive, bacterial outcome	8.90%		
	Bacterial-viral coinfection detection rate	89.40%		

CAP, community-acquired pneumonia; MMBV, MeMed BV; SOC, standard of care.

MMBV and MMBV + SOC diagnostic accuracies were calculated from the reported over- and underuse of antibiotics in paediatric and adult prospective observational cohort studies.^15,16^ Overuse cases informed sensitivity and underuse cases informed specificity. The obtained diagnostic accuracy parameters along with the infectious aetiology distribution in CAP were used to calculate true positive, true negative, false positive and false negative rates for the different populations. These calculations are summarized below:

- True positive rate: % bacterial infection × sensitivity [correct bacterial diagnosis].
- False negative rate: % bacterial infection × (1 − sensitivity) [bacterial infection not diagnosed].
- False positive rate: % viral infection × (1 − specificity) [viral infection not diagnosed].
- True negative rate: % viral infection × specificity [correct viral diagnosis].

For the scenario of bacterial-viral coinfections, reported data on viral PCR-positive cases with bacterial infectious aetiology were used.^15,16^

#### Cost and resource use

Cost inputs were divided into four categories: diagnostic testing, antibiotic drug acquisition, hospital admission due to misdiagnosis, and hospital readmission due to misdiagnosis. The baseline inputs on cost and resource use are summarized in Table 2.

**Table 2.** Base case cost and resource use inputs

I. Diagnostic test costs and resource use					
Parameter	Unit cost	Source	Resource use (Yes = 1; No = 0: [% patients])		Source
			Adults	Children	
Sputum test	£9	DAPS05^27^	1 [25.0]	0 [0]	Assumption; see Methods
Chest X-ray	£41	Imaging PF^27^	1 [100]	1 [100]	Assumption; see Methods
C-reactive protein (CRP)	£13	NIHR code:^28^ 86140	1 [69.7]	1 [62.7]	Assumption; see Methods
Rapid PCR panel	£50	NIHR code:^28^ 87798	1 [41.7]	1 [41.7]	Assumption; see Methods
Urinalysis	£20	NIHR code:^28^ 85027	1 [14.7]	1 [14.7]	Assumption; see Methods
Complete blood count (CBC)	£13	NIHR code:^28^ 86140	1 [90.2]	1 [97.6]	Assumption; see Methods
Procalcitonin (PCT)	£66	NIHR code:^28^ INV 156	1 [50.0]	1 [50.0]	Assumption; see Methods
MMBV	£65	Provided by MeMed	1 [100]	1 [100]	Assumption; see Methods

II. Antibiotic decision making						
IIa. Treatment unit cost						
Parameter	mg/capsule	Pack size	Price/pack	Numbers of capsules per day		Source
				Adults	Children	
Amoxicillin	500 mg	21 tablets	£0.21	3 tablets	3 tablets	^29^

IIb Treatment length (per episode)						
Parameter		Number of days		% Patients		Source
		Adults	Children	Adults	Children	
Correct bacterial diagnosis		8.0	8.0	100	100	^26^ Assumption: see Methods
Correct viral diagnosis		0	0	0	0	
Diagnosed viral but have bacterial		0	0	0	0	
Diagnosed bacterial but have viral		12.0	12.0	100	100	
						

III. Hospital admission due to misdiagnosis			
Parameter	Adults	Children	Source
Diagnosed viral but have a bacterial infection			
Proportion of patients who require admission	20%	20%	Assumption; see Methods
Total cost per admission	£3904		^30^
Diagnosed bacterial but have a viral infection			
Time spent as an inpatient	53.9 h	53.9 h	^31^
Cost per excess bed day	£275		^32^
Cost per hour	£11.46		

IV. Hospital readmission due to misdiagnosis			
Parameter	Adults	Children	Source
Proportion of patients who require readmission	10%	10%	Assumption: see Methods
Total cost per readmission	£3904		^30^

MMBV, MeMed BV; NIHR, National Institute for Health and Care Research.

No discount rates were applied. In the SOC + MMBV arm, cost and resource use associated with the MMBV test were applied to all patients that required testing following the clinician’s decision. Those with an equivocal MMBV score were assumed to incur the cost of further diagnostic tests. In the SOC arm, the subgroup with a diagnostic workup would receive the additional diagnostic tests. No data were available to precisely define the number of patients receiving each of the additional diagnostic tests. Therefore, in the base case analysis, it was assumed that all patients with further diagnostic testing would incur the diagnostic tests according to the frequency of resource use listed in Table 2. The frequency of diagnostic test resource use was informed by the interviews in the CPA, input from independent clinicians and unpublished evidence (provided by MeMed). Incorporating the frequency of diagnostic test use aligns with findings from previous MMBV studies, which highlight the variable availability of different diagnostic tests in informing clinical practice.^15–17^ Unit costs related to diagnostic testing were sourced from the National Cost Collection and Interactive costing tool (iCT) published (2023) by the UK National Institute for Health and Care Research (NIHR).^27,28^ The unit cost for the MMBV test was provided by MeMed.

The unit cost for antibiotics was sourced from the electronic market information tool (eMIT; 2023).^29^ Hospital admission and readmission resource use and costs were based on the cost per excess bed day for pneumonia in 2023 (HRG code DZ23). The additional time spent in hospital for patients incorrectly diagnosed with a bacterial infection was reported for a paediatric patient cohort from the UK.^31^ The additional time spent as inpatients arose from admission to observational wards to ensure the patient’s condition did not deteriorate. It was assumed that a proportion of patients incorrectly diagnosed with a viral infection would require hospital admission following the initial misdiagnosis. No data were available, so a conservative base case analysis was set.

### Deterministic sensitivity analysis

To account for first-order uncertainty around the data used for all input parameter values, all efficacy and resource use parameters were included in deterministic sensitivity analysis (DSA). The main output from the DSA was a tornado diagram. Where possible, the ranges applied to each parameter were based on SDs and 95% CIs. For parameters in which uncertainty data were not available, ranges of ±25% were applied around the point estimate that was applied in the base case analysis. The economic analysis and reporting were in line with reporting guidance for health economic evaluations.^33^

## Results

### Care pathway analysis

Eleven clinicians and three budget holders were interviewed between July and August 2023 (Table S1). All interviewees were based in the UK and had a median experience of 20 years (range: 2 to 38). Four key themes were identified: (i) care pathway for suspected LRTI, (ii) introduction of the MMBV test, (iii) adoption of MMBV in the NHS, and (iv) procurement in the NHS. To inform the economic model, Themes 1 and 2 were converted into a flowchart that captured the main decision points in the current diagnostic pathway for LRTI (which covers the diagnosis of CAP). The outcome SOC and adjusted SOC + MMBV clinical pathways (Figures S1 and S2) were used to inform the economic decision trees (Figure 1). The main points of deviation identified between hospitals were the time of blood draw and availability of diagnostic point-of-care tests. Clinician recommendations on the MMBV test regarding unmet clinical needs, MMBV test positioning and potential barriers to adoption were used to inform economic drivers for the model (as described in Methods).

### Base case analysis

The main clinical and cost outcomes are provided in Tables 3 and 4 and represent the comparison between the model decision trees for the cohort of 1000 patients with mild/moderate illness severity (adults and children modelled independently). The overall integration of the MMBV test in the clinical pathway for suspected CAP patients was estimated to provide total cost savings of £134 018 and £105 750 for adults and children, respectively.

**Table 3.** Clinical analysis in the base case

	SOC		SOC + MMBV		ΔSOC − (SOC + MMBV)	
	Adults	Children	Adults	Children	Adults	Children
**Overall diagnosis, *n***						
Correct viral diagnosis	302	629	471	752	169	123
Correct bacterial diagnosis	312	97	312	98	0	1
**Clinical parameters, *n***						
Total antibiotic use^a^	692	362	523	241	169	121
Other diagnostic tests	668	668	68	66	600	602
Hospital admission	381	267	212	144	169	123
Readmissions	1	1	1	1	0	0

MMBV, MeMed BV; SOC, standard of care.

^a^Total antibiotic use refers to the number of patients receiving antibiotic treatment overall, which is the sum of correct bacterial diagnosis and viral not diagnosed groups.

**Table 4.** Cost analysis in the base case

	SOC		SOC + MMBV		ΔSOC − (SOC + MMBV)	
	Adults	Children	Adults	Children	Adults	Children
**Total cost, £**						
Full cohort	323 675	254 281	189 658	148 531	134 018	105 750
Per person	324	254	190	149	134	106
**Cost per person, by patient group, £**						
Correct bacterial diagnosis	26	8	16	5	9	3
Correct viral diagnosis	24	50	29	42		8
Bacterial not diagnosed	8	11	7	10	0	2
Viral not diagnosed	266	185	137	92	129	93
**Cost per person, by resource type, £**						
MMBV test	—	—	43	43	−43	−43
Other diagnostic tests	81	79	8	8	73	72
Antibiotic treatment	0.98	0.55	0.70	0.35	0.28	0.20
Hospital admission	239	171	135	94	104	77
Readmission	2	4	2	4	0	1

MMBV, MeMed BV; SOC, standard of care.

Based on the data applied in the model, it was predicted that of the cohort of 1000 patients, 682 and 894 adults and children, respectively, would have a viral infection (with the remaining patients having a bacterial infection). In the SOC + MMBV arm of the model, a higher number of patients were correctly diagnosed as having a viral infection for both adults (471/1000 compared with 302/1000) and children (752/1000 compared with 629/1000). The difference in outcomes is associated with the improved specificity of the SOC + MMBV arm. The difference in the accuracy of detecting a bacterial infection was either negligible (children) or zero (adults). The impact in the changed correct/missed diagnosis of all infections resulted in:

- Reduction in total antibiotic treatment from 692/1000 to 523/1000 and from 362/1000 to 240/1000 for adults and children, respectively.
- Reduction in the number of patients receiving additional diagnostic tests from 668/1000 to 68/1000 and from 668/1000 to 66/1000 for adults and children, respectively.
- Reduction in hospital admissions from 381/1000 to 212/1000 and from 267/1000 to 144/1000 admissions for adults and children, respectively.

There was no change in the readmission rate. The improved viral infection diagnosis was the major driver for cost savings, with minor savings estimated for the correct bacterial diagnosis, correct viral diagnosis, and bacterial misdiagnosis group (Table 4). Overall, the cost benefit in other areas offset the added cost per person for the MMBV test resulting in total per patient savings of £134 (adults) and £106 (children) following the introduction of the MMBV test.

### Deterministic sensitivity analysis

**Figure 2.**
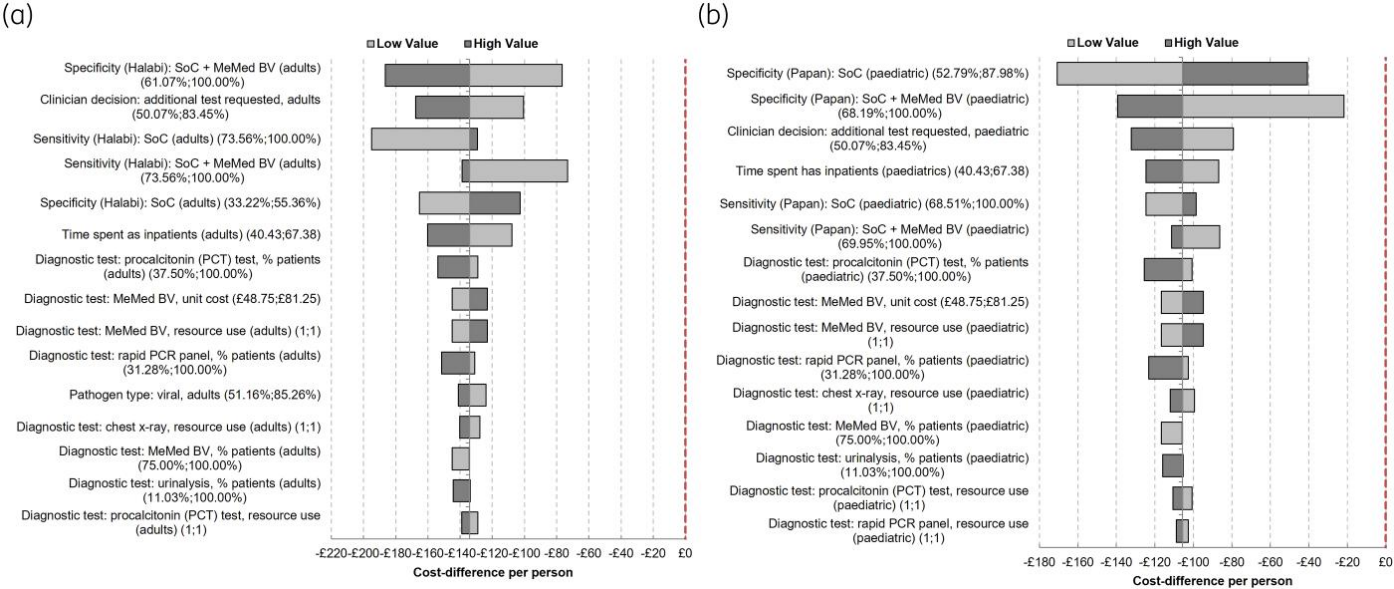
Tornado diagram showing the results of the deterministic sensitivity analysis. The results show the cost difference per person of the SOC + MMBV strategy compared with SOC alone, plotted for the adult (a) and paediatric (b) populations, using specificity and sensitivity data from Halabi *et al.*^16^ and Papan *et al.*^15^, respectively. None of the inputs varied within the tornado diagram caused the SOC + MMBV strategy to become more costly when compared with SOC alone. The dashed line represents the cost neutrality line (£0).

To account for first-order uncertainty in the data used for all input parameters, DSA was performed. A tornado diagram (Figure 2) was plotted and the 15 parameters with the greatest impact on cost savings displayed. For the adult subgroup (Figure 2a), when all other inputs remained constant, the specificity of SOC + MMBV and clinical decision of whether tests were required were the primary drivers of the cost model. Within the uncertainty ranges of the DSA, potential cost savings varied between £76 (low value) and £186 (high value) for the primary cost driver, i.e. specificity SOC + MMBV. For the paediatric subgroup (Figure 2b), the specificity of SOC and of SOC + MMBV were the primary drivers of the cost model. Within the uncertainty ranges of the DSA, potential cost savings varied between £170 (low value) and £40 (high value) for the primary cost driver, i.e. specificity SOC.

Importantly, none of the inputs varied within the tornado diagram caused the SOC + MMBV to become more costly when compared with SOC alone in both the adult and paediatric populations. In addition, the frequency of use of different diagnostics had a minor impact on the model outcome when compared with the primary cost driver for both adults and children (Figure S3). Overall, the potential cost savings for all parameters assessed in the DSA fluctuated between a low of £180 and a high of £186, and a low of £20 and a high of £140 for the adult and paediatric populations, respectively.

### Coinfection scenario

A scenario analysis was conducted where patients with a bacterial-viral coinfection entered the model to be diagnosed by SOC alone or SOC + MMBV. The main cost outcomes for the coinfection scenario are provided in Table S3. The modelled cohort comprised 1000 patients with confirmed viral infection and a secondary bacterial infection rate of 11% and 9% for adults and children, respectively. The SOC + MMBV remained cost-saving compared with SOC alone when detecting coinfections. Overall, integration of the MMBV test in this scenario is estimated to provide total cost savings of £256 317 and £256 132 for adult and child cohorts of 1000 patients, respectively. In the cost breakdown by resource type, SOC + MMBV was more expensive in terms of cost associated with MMBV tests and treatment cost. However, these costs were offset by the savings associated with delayed treatment and the associated cost of hospitalization for patients that were not appropriately diagnosed as having a coinfection.

## Discussion

In the current study, we report an early cost-consequence model that estimated the cost difference of SOC + MMBV versus SOC alone for patients who presented to a UK emergency department with suspected CAP. The model predicts that the introduction of SOC + MMBV has the potential to be cost saving, while promoting antibiotic stewardship, for both adult and paediatric CAP. Primary cost drivers were improved diagnostic stewardship and admission avoidance.

Previous economic models have been developed for the MMBV test. A model for CAP patients in the USA identified an overall per-patient saving of $37 and $223 saving for providers.^34^ Similarly, a European model focusing on Italy, Spain and Germany identified potential cost savings of €364 and €328 for hospitals, and €91 and €59 for payers in Italy and Germany, respectively.^35^ In Spain, overall savings were estimated to be €165 per patient.^35^ However, there are important differences between the models. First, the current model was informed by a CPA that mapped the clinical decision-making process. This reduced the number of modelling assumptions ensuring the model structure was a more accurate representation of clinical practice. Furthermore, incorporating a CPA was recently recommended as the most accurate approach when assessing the cost-effectiveness of diagnostics.^19^ Second, the modelled cost drivers were different. Previous models focused on savings linked to antibiotic stewardship and adverse events. Our cost drivers for the model focused on diagnostic stewardship, antibiotic costs, admission avoidance and readmission costs. The current model did not identify a cost saving for antibiotics, as could be expected due to the low unit cost of antibiotics. Finally, the current model makes use of UK-specific unit costs for diagnostics and treatments, as well as costs associated with hospital stay.

This study has limitations. First, the model captures a simplified version of the actual CAP clinical pathway and, therefore, does not quantify all possible patient outcomes. The model assigned a correct bacterial diagnosis with oral antibiotics and discharge. In reality, patients may also receive IV antibiotics, be admitted to the ward or die. Second, cost savings from diagnosing bacterial infection earlier and preventing patient illness progression were not captured. Third, the resource use estimated within the model is based on the diagnostic accuracy of MMBV, as measured within prospective observational studies. Fourth, assumptions were required to fully populate the model. In particular, the rate of admission or readmission following the misdiagnosis of a bacterial infection. However, this assumption is accounted for in the DSA. Finally, the model was limited to CAP patients with a low to medium severity risk profile, omitting the benefit for patients with high severity profile. The low to medium patient group is likely to have a lower (re)admission rate, which may not be captured adequately in this model. Published evidence, however, indicates that the majority of hospitalized CAP patients do not have a high severity risk profile, reaffirming our base-case assumption.^4,5,31,36^ Furthermore, as there is currently no economic variable associated with antibiotic use and AMR, the low acquisition costs of the antibiotics in the model might underestimate the economic benefits of diagnostic interventions that reduce antibiotic use.

In summary, the current economic model supports the potential beneficial use of MMBV in the NHS based on its favourable cost-saving profile. When implemented, MMBV may aid both antibiotic and diagnostic stewardship.

## Supplementary Material

dlaf016_Supplementary_Data

## Supplementary data

Figures S1 to S3, Tables S1 to S3 and Appendix S1 are available as Supplementary data at *JAC-AMR* Online.
