# Supplementary material for: Host-response testing with MeMed BV in community-acquired pneumonia: an economic evaluation from the UK NHS perspective
Source: JAC Antimicrob Resist. 2025 Feb 19;7(1):dlaf016. doi: 10.1093/jacamr/dlaf016 (PMC11836884; doi:10.1093/jacamr/dlaf016)
Supplement: dlaf016_Supplementary_Data [file dlaf016_supplementary_data.docx]

**Host-Response Testing with MeMed BV in Community-Acquired Pneumonia: An Economic Evaluation from the UK NHS Perspective.**

**Supplemental Data**


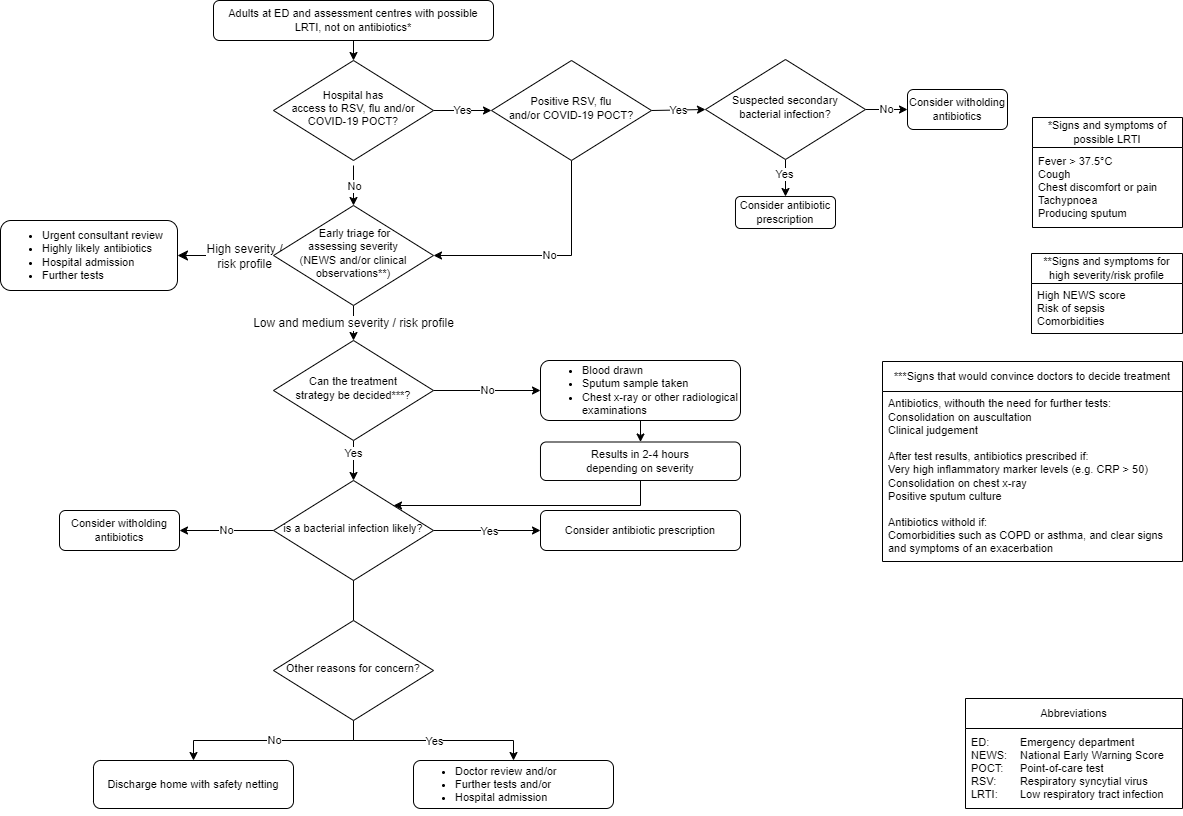


**Figure S1:** Clinical care pathway for SOC


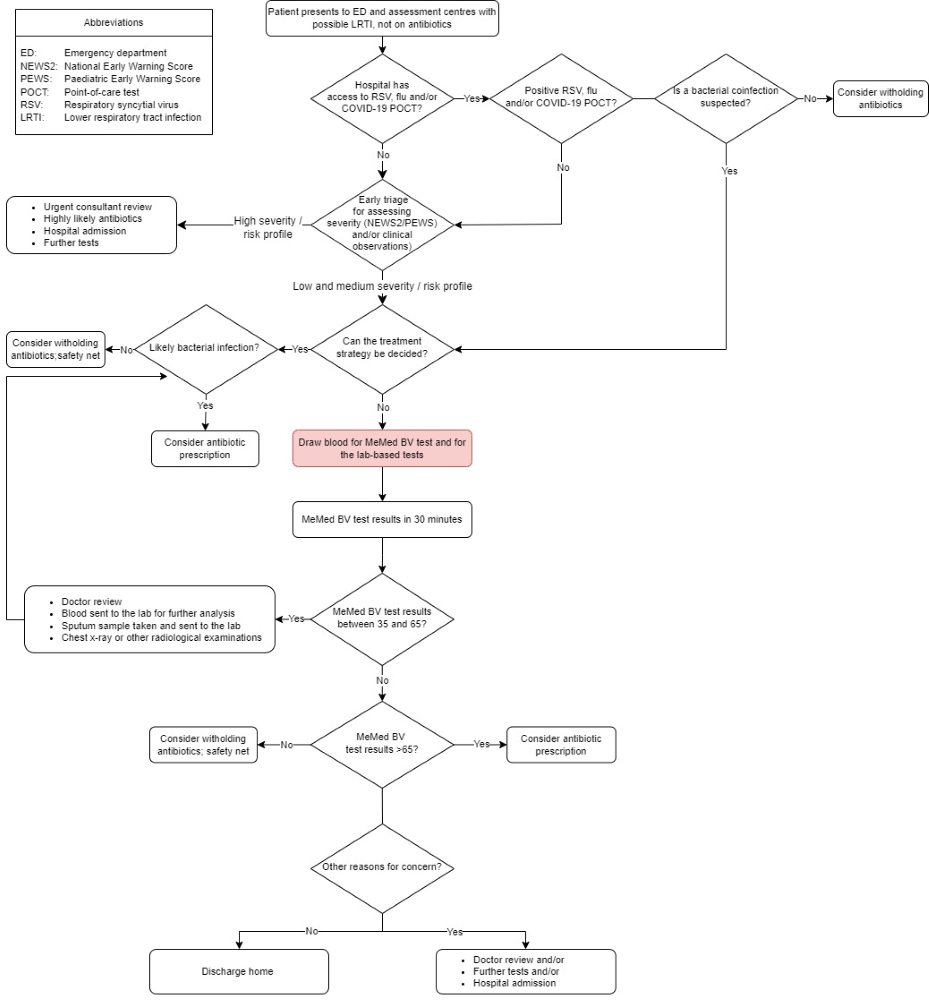


**Figure S2:** Clinical care pathway for SOC+MMBV


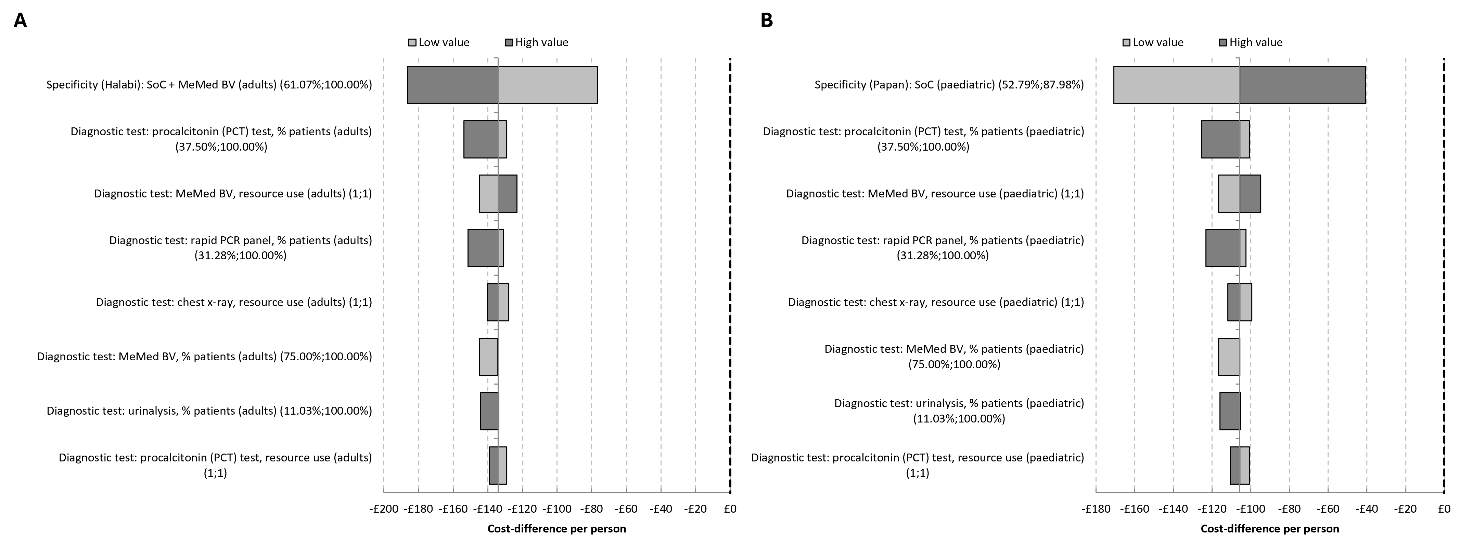


**Figure S3: Tornado diagram showing the results of the deterministic sensitivity analysis for different diagnostic tests utilized.** The results show the cost-difference per person of the SOC+MMBV strategy compared to SOC alone, plotted for the adult (A) and paediatric (B) population. The outcome is focused on frequency of use of different diagnostics tests compared to the primary cost driver in the model. The frequency of diagnostic test usage had a limited impact on the outcome of the model when compared to the primary cost driver. The dashed line represents the cost neutrality line (£0).

**Table S1:** Interviewee characteristics

| **Interviewee #** | **Topic Guide** | **Role** | **Experience (years)** |
| --- | --- | --- | --- |
| I1 | Budget stakeholder | Diagnostics Adoption Specialist | 28 |
| I2 | Clinician stakeholder | Consultant Paediatrician | 20 |
| I3 | Clinician stakeholder | Infectious Disease Consultant | 23 |
| I4 | Clinician stakeholder | Respiratory Medicine Consultant | 20 |
| I5 | Clinician stakeholder | Infectious Disease Consultant | 20 |
| I6 | Clinician stakeholder | Consultant microbiologist | 12 |
| I7 | Clinician stakeholder | Consultant in Acute Medicine | 29 |
| I8 | Budget stakeholder | Procurement Manager | 2 |
| I9 | Clinician stakeholder | Consultant Microbiologist | 12 |
| I10 | Clinician stakeholder | Emergency Medicine Consultant | 19 |
| I11 | Budget stakeholder | Consultant Biochemistry | 38 |
| I12 | Clinician stakeholder | Paediatric Emergency Medicine Consultant | 21 |
| I13 | Clinician stakeholder | Paediatric Respiratory Medicine Consultant | 20 |
| I14 | Clinician stakeholder | Respiratory Medicine Consultant | 24 |

**Table S2: Completed CHEERS criteria checklist.**

| **Topic** | **No.** | **Item** | **Location where item is reported** |
| --- | --- | --- | --- |
| **Title** | | | |
|  | 1 | Identify the study as an economic evaluation and specify the interventions being compared. | Page 1 |
| **Abstract** | | | |
|  | 2 | Provide a structured summary that highlights context, key methods, results, and alternative analyses. | Page 2 |
| **Introduction** | | | |
| Background and objectives | 3 | Give the context for the study, the study question, and its practical relevance for decision making in policy or practice. | Page 3-4 |
| **Methods** | | | |
| Health economic analysis plan | 4 | Indicate whether a health economic analysis plan was developed and where available. | Page 5-6 |
| Study population | 5 | Describe characteristics of the study population (such as age range, demographics, socioeconomic, or clinical characteristics). | Page 6 |
| Setting and location | 6 | Provide relevant contextual information that may influence findings. | Page 5-6 |
| Comparators | 7 | Describe the interventions or strategies being compared and why chosen. | Page 5-7 |
| Perspective | 8 | State the perspective(s) adopted by the study and why chosen. | Page 6 |
| Time horizon | 9 | State the time horizon for the study and why appropriate. | Page 6 |
| Discount rate | 10 | Report the discount rate(s) and reason chosen. | Page 8 |
| Selection of outcomes | 11 | Describe what outcomes were used as the measure(s) of benefit(s) and harm(s). | Page 6 |
| Measurement of outcomes | 12 | Describe how outcomes used to capture benefit(s) and harm(s) were measured. | Page 6 |
| Valuation of outcomes | 13 | Describe the population and methods used to measure and value outcomes. | Page 6-9 |
| Measurement and valuation of resources and costs | 14 | Describe how costs were valued. | Page 8-9 |
| Currency, price date, and conversion | 15 | Report the dates of the estimated resource quantities and unit costs, plus the currency and year of conversion. | Page 9 |
| Rationale and description of model | 16 | If modelling is used, describe in detail and why used. Report if the model is publicly available and where it can be accessed. | Page 6-9 |
| Analytics and assumptions | 17 | Describe any methods for analysing or statistically transforming data, any extrapolation methods, and approaches for validating any model used. | Not applicable |
| Characterising heterogeneity | 18 | Describe any methods used for estimating how the results of the study vary for subgroups. | Page 7 |
| Characterising distributional effects | 19 | Describe how impacts are distributed across different individuals or adjustments made to reflect priority populations. | Page 8 |
| Characterising uncertainty | 20 | Describe methods to characterise any sources of uncertainty in the analysis. | Page 9 |
| Approach to engagement with patients and others affected by the study | 21 | Describe any approaches to engage patients or service recipients, the general public, communities, or stakeholders (such as clinicians or payers) in the design of the study. | Page 5 |
| **Results** | | | |
| Study parameters | 22 | Report all analytic inputs (such as values, ranges, references) including uncertainty or distributional assumptions. | Page 10-12 and 23-29 |
| Summary of main results | 23 | Report the mean values for the main categories of costs and outcomes of interest and summarise them in the most appropriate overall measure. | Page 10-12 and 23-29 |
| Effect of uncertainty | 24 | Describe how uncertainty about analytic judgments, inputs, or projections affect findings. Report the effect of choice of discount rate and time horizon, if applicable. | Page 11-12 and 23 |
| Effect of engagement with patients and others affected by the study | 25 | Report on any difference patient/service recipient, general public, community, or stakeholder involvement made to the approach or findings of the study | Not applicable |
| **Discussion** | | | |
| Study findings, limitations, generalisability, and current knowledge | 26 | Report key findings, limitations, ethical or equity considerations not captured, and how these could affect patients, policy, or practice. | Page 13-15 |
| **Other relevant information** | | | |
| Source of funding | 27 | Describe how the study was funded and any role of the funder in the identification, design, conduct, and reporting of the analysis | Page 16 |
| Conflicts of interest | 28 | Report authors conflicts of interest according to journal or International Committee of Medical Journal Editors requirements. | Page 16 |

*From: Husereau D, Drummond M, Augustovski F, et al. Consolidated Health Economic Evaluation Reporting Standards 2022 (CHEERS 2022) statement: updated reporting guidance for health economic evaluations. BMJ. Published online January 11, 2022:e067975. doi:10.1136/bmj-2021-067975*

**Table S3:** Cost analysis for the co-infection scenario

|  | **SOC** | | **SOC+MMBV** | | **ΔSOC - (SOC+MMBV)** | |
| --- | --- | --- | --- | --- | --- | --- |
|  | Adults | Children | Adults | Children | Adults | Children |
| Total cost | | | | | | |
| Full cohort | £442,745 | £356,092 | £82,389 | £101,919 | £360,356 | £254,174 |
| Per person | £443 | £356 | £82 | £102 | £360 | £254 |
| Cost per person, by patient group | | | | | | |
| Co-infection | £443 | £356 | / | / | £443 | £356 |
| Co-infection – detected | / | / | £7 | £5 | -£7 | -£5 |
| Co-infection – not detected | / | / | £18 | £37 | -£18 | -£37 |
| No co-infection | / | / | £58 | £59 | -£58 | -£59 |
| Cost per person, by resource type | | | | | | |
| MMBV test | / | / | £65 | £65 | -£65 | -£65 |
| Other diagnostic tests | £11 | £9 | / | / | £11 | £9 |
| Antibiotic treatment | / | / | £0 | £0 | £0 | £0 |
| Hospital admission | £432 | £347 | £17 | £37 | £415 | £311 |

Abbreviations: MMBV – MeMed BV; SOC – standard of care.

**Appendix S1: Topic Guide for ‘clinician’ interviews**

Introduction (5 minutes)

- Thank the interviewee for agreeing to take part.
- Give a brief introduction to the York Health Economics Consortium (YHEC) and its role in the project:
  - YHEC is a health economic consulting company owned by the University of York. YHEC provides national and international consultancy in health economics and outcomes research to the NHS, pharmaceutical and healthcare industries.
  - A medical device company has commissioned YHEC to undertake a care pathway analysis of a new test to support clinical decision making in patients with suspected community acquired pneumonia (CAP).
- Remind the interviewee that:
  - The interview is scheduled for 60 minutes and will be recorded (as per the consent form).
  - They should not disclose any personal information or confidential patient information during the interview (other than when asked about their role / previous experience).
  - Any accidental disclosure of confidential information will not be transcribed and will be deleted from the audio recording.
- Explain that we will begin the interview with some questions about their role/experience before asking questions about the current care pathway and then focussing on the new test.
- Check if the interviewee has any questions before starting the interview.
- Start the recording in Zoom.

Background Questions (5 minutes)

Remind the interviewee not to mention their name when answering the background questions:

1. What is your role and speciality? Which department do you work in? A&E, ICU, ward or laboratory?
2. Which hospital/organisation and city/region do you work in?
3. Do you work with adults and children?
4. Does your hospital have an onsite laboratory or is it external?
5. How many years of relevant experience do you have?
6. 6. Do you have experience with the adoption of new tests in your hospital?
   - If yes, can you briefly describe the adoption pathway for a new test in your hospital?
   - Who are the important decision makers in this adoption pathway?
   - Do you have any advice for test developers who are aiming for adoption in the NHS?

Care Pathway (15 to 20 minutes)

- Ask if the interviewee had the opportunity to read through the material that was shared in advance of the interview.
- Explain that we will now look at the draft care pathway diagram which shows our understanding of the clinical decisions involved when a patient presents to secondary care with suspected CAP in the UK.
- Explain that our draft pathway was developed based on the NICE, British Thoracic Society and British Society for Antimicrobial Chemotherapy guidelines for suspected CAP. Explain that while the guidelines represent what should be happening, there is often uncertainty and discrepancy in practice depending on the disease. What we want to understand is what is actually happening in clinical practice and where there is variability in the pathway for suspected CAP in the UK.
- Clarify that we are looking for feedback on the pathway based on the clinician’s experience with adults or children who present with suspected CAP to help us understand if the pathway aligns with clinical practice and if anything is missing from the diagram:
  - If we are interviewing a paediatrician, we will only ask questions relating to children, while if they specialise in adult patients, we will focus the questions on adults. If they treat both children and adults, we will focus on adults but include a question at the end about major differences for children (or vice versa depending on how many paediatricians we can recruit).

Questions

1. In your experience, what proportion of patients with fever / signs and symptoms suggestive of CAP tend to present in A&E on antibiotics already? If they are on antibiotics already, do you ever stop them if you suspect a viral infection, or do you tend to finish the course? What could influence this choice?
2. What factors influence your decision to prescribe or not prescribe antibiotics (patient/disease characteristics e.g. comorbidities, clinical severity)? Do you always prescribe antibiotics if you have strong evidence of bacterial infection? And do you always not prescribe them if you suspect a viral infection?
3. How do you decide if the infection is viral or bacterial? Which tests / clinical observations do you use? When?
4. Would you always draw blood from patients with suspected CAP? If not, how would you decide whether to draw blood or not? When do you draw the blood?
5. Who does take the decision of starting/stopping antibiotics and admitting or not a patient to the ward?
6. What are the consequences if a patient is incorrectly diagnosed as having a viral or bacterial infection? Would these patients require additional resources compared with those who are correctly diagnosed?
7. The NICE and BTS guidelines state that adults with CAP who require antibiotic treatment should receive it within four hours of presentation at A&E. How often is this achieved?
8. What are the unmet clinical needs in the current pathway? What are the biggest delays before antibiotics are prescribed?

Optional additional questions

1. Is the diagnosis of CAP usually confirmed with a chest X-ray?

**Test Description (25 to 30 minutes)**

Evidence

As of April 2023, the test has been included in eight diagnostic studies summarised below.

Index test:

- The serum concentrations of the three host proteins (TRAIL, CRP and IP-10) were measured and computationally integrated into a single score.
- Patients with an index test score below 0.35 are classified as viral, between 0.35 and 0.65 as equivocal, and above 0.65 as bacterial (as shown in the image above).
- Early clinical validation was performed using the first-generation manual version of the test (with the same biomarkers). The FDA and MHRA approved that this evidence can be transferred to the automated version of the test.

Reference standard:

- Given the absence of a gold standard for determining bacterial versus viral aetiology, the reference standard was generated based on expert panel adjudication with three clinicians.
- Mixed infections (bacterial and viral) were labelled as bacterial because they are managed similarly.

Questions

Clarify that the next interview questions are focused on the whole-blood version of the new test in a population of adults or children with suspected CAP:

1. Where do you think the new test could fit into the current pathway? Could it have more than one role/position in the pathway? Could this test benefit the healthcare system, clinicians and patients? Where in the pathway most? Which patients would benefit most from the new test (severity of CAP, any other factors)?
2. Do you think introduction of the new test could have a negative impact on the pathway for suspected CAP? In what way?
3. We think the test could be introduced when triaging the patient (before CAP is confirmed), do you agree with this? What would be the implications of this on your decision making?
4. In which proportion of patients would it not be acceptable to wait 15 to 30 minutes for the test results before prescribing antibiotics? In which situations?
5. How difficult/easy do you think it would be to introduce this test into the CAP pathway? What are potential problems when introducing it?
6. What are the potential facilitators to adoption of the test in the UK? Can you think to an example of a test that was successfully introduced into the NHS? What did support its adoption?
7. Do you think approval from NICE would help facilitate adoption of this test or is robust evidence enough? Do you think more evidence is needed to support adoption of the test?
8. Do you know how to get a new test like this approved in your hospital?
9. Do you think the test could be useful in another population other than CAP? If you had to choose one population where this test could be most useful and has the most unmet need, what would it be?
10. Would there be any role or advantage to have such a diagnostic test available using a serum-based sample on a larger automatic diagnostic platform in the central laboratory (as part of the laboratory 24/7 track system)?

Optional additional questions

1. Do you think the test would be useful for patients who have presented on antibiotics? How? Would you stop antibiotics if the test indicated that a viral infection is likely?
2. Are you aware of any major variations in the CAP pathway across the UK?
3. If implemented, where would the best (and most practical) location for the device be in your hospital? How do you think the test would be run in your hospital? For example, would the sample go to the lab or be conducted by a nurse?

**Final Comments (5 minutes)**

1. Are there any final comments you would like to add?

- Stop and save the recording in Zoom.
- Thank the interviewee for participating in the project.
- Remind the interviewee that we will produce a summary of the interview that we will ask them to check for accuracy.
